# Supplementary material for: Maternal Dietary Selenium Intake during Pregnancy Is Associated with Higher Birth Weight and Lower Risk of Small for Gestational Age Births in the Norwegian Mother, Father and Child Cohort Study
Source: Nutrients. 2020 Dec 23;13(1):23. doi: 10.3390/nu13010023 (PMC7822440; doi:10.3390/nu13010023)
Supplement: Supplementary file 1 [file nutrients-13-00023-s001.pdf]

Supplementary tables

Table S1: Associations between maternal dietary selenium intake and infant birth weight percentile according to ultrasound-based and customized growth standards in women in the Norwegian Mother, Father and Child Cohort study

	Birth weight percentile according to:							
	**Ultrasound-based growth curves**				**Customized growth curves**			
	**n**	**ß² (95% CI)**	**SE**	**P**	**n**	**ß² (95% CI)**	**SE**	**P**
Unadjusted	71,703	1.0 (0.8, 1.2)	0.1	5.9×10^{-20}	70,767	0.7 (0.5, 0.9)	0.1	5.9×10^{-11}
Adjusted[1]	66,898	0.7 (0.3, 1.1)	0.2	8.2×10^{-4}	66,071	0.7 (0.3, 1.1)	0.2	5.2×10^{-4}

Multiple linear regression analysis of standardized selenium intake from food in relation to birth weight percentile according to ultrasound-based [38] and customized [39] growth standards.
[1]Adjusted for maternal age at delivery, maternal pre-pregnancy body mass index (BMI), parity, maternal smoking during pregnancy, passive smoking, nausea during second trimester, maternal education, fibre intake, iodine intake, protein intake, n-3 intake from diet and total energy intake.
[2]ß per SD of selenium intake in µg/day
The adjusted models included fewer women due to missing data on the covariates.
Abbreviations: ß - beta, CI – confidence interval, SE – standard error.

Table S2: Associations between maternal dietary selenium intake and infant small for gestational age (SGA) status according to ultrasound-based and customized growth standards women from the Norwegian Mother, Father and Child Cohort study

	SGA definition according to					
	**Ultrasound-based growth curves**			**Customized growth curves**		
	**n total/SGA**	**OR² (95% CI)**	**P**	**n total/SGA**	**OR² (95% CI)**	**P**
Unadjusted	71,703/ 1,422	0.95 (0.90, 0.99)	<0.05	70,767/ 10,237	0.95 (0.93, 0.97)	7.8×10^{-6}
Adjusted[1]	66,898/ 1,323	0.93 (0.84, 1.04)	0.199	66,071/9,532	0.95 (0.91, 0.99)	0.020

Multiple logistic regression analysis of standardized selenium intake from food in relation to percentiles of birth weight according to ultrasound-based [38] and customized [39] growth standards.
[1]Adjusted for maternal age at delivery, maternal pre-pregnancy body mass index (BMI), parity, maternal smoking during pregnancy, passive smoking, nausea during second trimester, maternal education, fiber intake, iodine intake, protein intake, n-3 intake from diet and total energy intake.
[2]OR per SD of selenium intake in µg/day
The adjusted models included fewer women due to missing data on the covariates.
Abbreviations: CI – confidence interval, OR – odds ratio, SGA – small for gestational age.

Table S3: Associations between maternal selenium intake form supplements and infant birth weight percentile according to ultrasound-based and customized growth standards

		Birth weight percentile according to:							
		Ultrasound-based growth curves				Customized growth curves			
		**n**	**ß² (95% CI)**	**SE**	**P**	**n**	**ß² (95% CI)**	**SE**	**P**
Organic selenium	Unadjusted	71,698	-0.12 (-0.33, 0.09)	0.11	0.269	70,763	-0.05 (-0.26, 0.16)	0.11	0.637
	Adjusted¹	66,898	-0.02 (-0.23, 0.19)	0.11	0.864	66,071	-0.05 (-0.26, 0.17)	0.11	0.683
Inorganic selenium	Unadjusted	71,698	-0.23 (-0.44, -0.02)	0.11	0.035	70,763	0.20 (-0.01, 0.41)	0.11	0.056
	Adjusted¹	66,898	0.68 (0.20, 1.16)	0.68	0.005	66,071	0.83 (0.34, 1.32)	0.83	8.7×10^{-4}

Multiple linear regression analysis of standardized selenium intake from supplements in relation to birth weight percentile according to ultrasound-based [38] and customized [39] growth standards.

¹Adjusted for maternal dietary selenium intake, maternal age at delivery, maternal pre-pregnancy body mass index (BMI), parity, maternal smoking during pregnancy, passive smoking, nausea during second trimester, maternal education, fibre intake, iodine intake, protein intake, n-3 intake from diet and total energy intake.

²ß per SD of selenium intake in µg/day

The adjusted models included fewer women due to missing data on the covariates.

Abbreviations: ß - beta, CI – confidence interval, SE – standard error.

Table S4: Associations between maternal selenium intake from supplements and small for gestational age according to ultrasound-based and customized growth standards

		SGA definition according to					
		Ultrasound-based growth curves			Customized growth curves		
		**n total/SGA**	**OR² (95% CI)**	**P**	**n total/SGA**	**OR² (95% CI)**	**P**
Organic selenium	Unadjusted	71.698/ 1,422	1.04 (1.00, 1.08)	0.034	70,763/ 10,237	1.00 (0.98, 1.02)	0.764
	Adjusted¹	66,898/ 1,323	1.04 (1.00, 1.08)	0.032	66,071/ 9,532	1.00 (0.98, 1.02)	0.810
Inorganic selenium	Unadjusted	71,698/ 1,422	1.01 (0.95, 1.06)	0.816	70,763/ 10,237	0.99 (0.97, 1.01)	0.285
	Adjusted¹	66,898/ 1,323	0.98 (0.93, 1.03)	0.425	66,071/ 9,532	0.98 (0.96, 1.01)	0.145

Multiple logistic regression analysis of standardized selenium intake from food in relation to percentiles of birth weight according to ultrasound-based and customized growth standards.

¹Adjusted for maternal dietary intake of selenium, maternal age at delivery, maternal pre-pregnancy body mass index (BMI), parity, maternal smoking during pregnancy, passive smoking, nausea during second trimester, maternal education, fibre intake, iodine intake, protein intake, n-3 intake from diet and total energy intake.

²OR per SD of selenium intake from supplements in µg/day

The adjusted models included fewer women due to missing data on the covariates.

Abbreviations: CI – confidence interval, OR – odds ratio, SGA – small for gestational age.

Table S5: Associations between maternal whole blood selenium concentrations and birth weight percentile according to ultrasound-based and customized growth standards in women from the Norwegian Mother, Father and Child Cohort study

	**Birth weight percentiles according to:**							
	**Ultrasound-based growth curves**				**Customized growth curves**			
	**n**	**ß² (95% CI)**	**SE**	**P**	**n**	**ß² (95% CI)**	**SE**	**P**
Unadjusted	2628	-1.6 (-2.7, -0.52)	0.56	0.0037	2587	-0.70 (-1.8, 0.39)	0.56	0.206
Adjusted¹	2488	-0.60 (-1.7, 0.51)	0.56	0.289	2450	-0.31 (-1.5, 0.84)	0.59	0.600

Multiple linear regression analysis of log transformed selenium concentrations in maternal whole blood collected during pregnancy in relation to percentiles of birth weight according to ultrasound-based and customized growth standards.

[1]Adjusted for maternal age at delivery, maternal pre-pregnancy body mass index (BMI), parity, maternal smoking during pregnancy, passive smoking and maternal education.

[2]ß per % of blood selenium

The adjusted models included fewer women due to missing data on the covariates.

Abbreviations: ß - beta, CI – confidence interval, SE – standard error.

Table S6: Associations between maternal selenium concentrations in whole blood and infant small for gestational age (SGA) status according to ultrasound-based and customized definitions in 2,628 women in the Norwegian Mother, Father and Child Cohort study

	**SGA definition according to**					
	**Ultrasound-based growth curves**			**Customized growth curves**		
	**n total/SGA**	**OR² (95% CI)**	**P**	**n total/SGA**	**OR² (95% CI)**	**P**
Unadjusted	2628/ 32	0.36 (0.07, 1.95)	0.243	2587/309	1.1 (0.96, 1.2)	0.179
Adjusted¹	2488/ 30	0.37 (0.06, 2.2)	0.282	2450/293	1.1 (0.93, 1.2)	0.442

Multiple logistic regression of log transformed maternal selenium concentrations in whole blood collected during pregnancy in relation to small for gestational age according to ultrasound-based and customized growth standards..

[1]Adjusted for maternal age at delivery, maternal pre-pregnancy body mass index (BMI), parity, maternal smoking habits during pregnancy, passive smoking and maternal education.

[2]OR per % of blood selenium

The adjusted models included fewer women due to missing data on the covariates.

Abbreviations: CI – confidence interval, OR – odds ratio, SGA – small for gestational age.
